# How do older adults receiving aged care services understand and respond to the EQ-5D-5L? A think-aloud study in residential care

**DOI:** 10.1007/s11136-023-03466-2

**Published:** 2023-06-29

**Authors:** Kiri Lay, Matthew Crocker, Lidia Engel, Julie Ratcliffe, Simon Charlton, Claire Hutchinson

**Affiliations:** 1 Health and Social Care Economics Group, Caring Futures Institute, Flinders University, GPO Box 2100, Adelaide, SA 5001 Australia; 2 Health Economics Division, School of Public Health and Preventive Medicine, Monash University, Melbourne, VIC Australia; 3 South Australian Innovation Hub, Adelaide, Australia

**Keywords:** Cognitive impairment, Long-term care, Older adults, EQ-5D, Think-aloud

## Abstract

**Purpose:**

The EQ-5D-5L is a preference-based instrument for measuring and valuing health-related quality of life (HRQoL). The EQ-5D-5L has been used extensively in economic evaluation, including in aged care. However, older adults’ understanding of the EQ-5D-5L has not been comprehensively investigated to date. This research aimed to assess older adults’ understanding of the EQ-5D-5L using a think-aloud protocol with two cognition groups: no cognitive impairment and mild/moderate cognitive impairment.

**Methods:**

Participants’ cognition was assessed using the Standardised Mini-Mental State Examination (SMMSE). Face-to face interviews were conducted with concurrent and retrospective think-aloud encouraged through verbal probing. Audio recordings were transcribed, and qualitative analysis, informed by the Tourangeau four-stage Response Model (comprehension, retrieval, decision process, response process) was conducted in NVivo.

**Results:**

In total, 46 older adults (age 65 +) were recruited from 10 residential care facilities across South Australia (*n* = 25 no cognitive impairment, *n* = 21 mild/moderate cognitive impairment). Comprehension, retrieval, judgement and response mapping issues were common across all cognition levels and EQ-5D-5L dimensions. The two dimensions resulting in the most response issues were usual activities and personal care.

**Conclusion:**

Older adults may bring a different understanding to the EQ-5D-5L descriptive system than that expected given testing with general population samples. Dimension descriptors that are more relevant to this population may facilitate responses that better align with the underlying EQ-5D-5L concept model.

**Supplementary Information:**

The online version contains supplementary material available at 10.1007/s11136-023-03466-2.

## Introduction

The global population is ageing rapidly. The proportion of older adults (people aged 65 years and over) is expected to increase from 9.7% in 2019 to 16.4% by 2050 worldwide [1]. Cognitive impairment and dementia are most prevalent in older age groups and commensurate with population ageing, the number of older adults diagnosed with dementia in Australia is predicted to more than double by 2058, [2]. Given population ageing and increasing resource scarcity, health and social care provision for older adults into the future will necessitate careful and proactive policy planning [3]. Economic evaluation of interventions and services for the care of older adults is necessary to ensure that funding is efficiently allocated to maximise older adults’ quality of life (QOL) [4]. Monitoring the health and wellbeing of older adults accessing care is also essential to ensure high quality service provision, as reflected by the recommendations of the recent Australian Royal Commission into Aged Care Quality and Safety [5]. Aged care in Australia is provided either through community home-care services, for people with low care needs or, for people requiring higher level of care and support, in residential care facilities. Whilst care needs vary, most residents (89%) have medium (26.8%) or high (62.6%) care needs in at least one domain of care [6].

Preference-based measures of QOL such as the EuroQol-5 Dimension-5 Level (EQ-5D-5L) instrument can be used for quality assessment and as a key outcome measure for the economic evaluation of health and social care interventions. For both quality assessment and economic evaluation, it is important to ascertain the person’s own assessment of their QOL wherever possible, hence self-reported QOL is preferable over proxy report [7, 8]. The EQ-5D-5L has been widely applied with older people in a variety of care settings including aged care [8, 9]. However, currently little evidence or practical guidance is available to the research community, policy makers and practitioners to indicate the validity of self-reported QOL using the EQ-5D-5L in populations of older people with varying levels of cognitive impairment and dementia.

Given that the EQ-5D-5L was developed with general population samples and not specifically with older adults, it is important to explore how older adults interpret and understand the EQ-5D-5L, and what information is being considered when responding to ensure that meaningful self-report data is being elicited [10]. This is particularly important for population of older people in aged care settings with a high prevalence of cognitive impairment and dementia.

Therefore, this study aimed to assess how older adults in residential aged care with different levels of cognitive impairment understand, interpret and respond to the EQ-5D-using a qualitative think-aloud approach. (background information on the think aloud approach is provided in Online Resource 1).

## Method

Ethical approval for this research was provided by the Flinders University Human Research Ethics Committee (Project Number: 6732).

### Participants

Participants were recruited through the South Australian Innovation Hub, a not-for-profit group of eight residential aged care providers. Target sample size guideline for cognitive interviewing studies indicate that 20 participants is sufficient to ensure data saturation [11]. Consenting older adults were stratified into one of two cognition sub-groups indicating the presence or absence of cognitive impairment with the aim of recruiting 20 participants in each sub-group for a total sample of 40 residents. Residents were eligible to participate if they could understand and communicate in the English language and were aged 65 or older. Residents with severe cognitive impairment or dementia who were unable to provide informed assent or consent to participate in the research were excluded [12].

### Materials

Interview Guide: A detailed interview guide (Online Resource 2) was developed based on published guidelines for cognitive interviewing [13].

Demographic questionnaire: Demographic questions included age, education level, country of birth and length of time residing at their residential facility.

Standardised Mini-Mental State Examination (SMMSE): The SMMSE is a standardised version of the Mini-Mental State Examination (MMSE), an internationally recognised screening tool for the assessment of cognitive impairment in older adults [14]. It consists of 12 items and scores range from 0 to 30. The UK’s National Institute for Health and Care Excellence (NICE) cut-off scores for cognition sub-group were utilised where a score of 10–20 indicates moderate cognitive impairment, 21–26 indicates mild cognitive impairment, and 27–30 indicates no cognitive impairment [15].

EuroQol-5 dimensions-5 level (EQ-5D-5L): The EQ-5D-5L was developed in 2009 from the original three-response level version (EQ-5D-3L) to improve the sensitivity and reliability of the instrument and to increase the number of possible health states [16]. The instrument consists of five dimensions: mobility, personal care, usual activities, pain/discomfort and anxiety/depression. Participants are asked to rate their health as of ‘today’, with five response options (no problems, slight problems, moderate problems, severe problems, extreme problems/unable). The EuroQoL visual analogue scale (EQ VAS) component of the EQ-5D-5L consists of a vertical scale numbered from 0 to 100, with 0 representing ‘the worst health you can imagine’ and 100 representing ‘the best health you can imagine’. Respondents are asked to choose a point on the scale that best represents their own health today.

### Procedure

Data were collected over a 6-month period from June to December 2021. Residential aged care facilities that expressed interest in the research were attended to by two of the authors who gave a short presentation about the research at a residents’ meeting and distributed participant information sheets and a project flier. Where the researchers were not able to attend a residents’ meeting (due to timing or COVID19 restrictions) fliers and participant information sheets were distributed by facility staff. Staff were informed that the research team sought participants with varying levels of cognitive decline and were explicitly asked to distribute information to all residents regardless of perceived capacity to participate or consent.

Contact details of interested residents were forwarded to the research team by the residential care facility’s manager following initial verbal consent. Days and times for interviews were co-ordinated with facility staff. Where residents had a power-of-attorney in place, assent was sought from the resident and consent from the person with power-of-attorney [17]. Participants were assessed and allocated into one of two cognition sub-groups, based on their score on the SMMSE.

Participants chose the interview location (e.g., their bedroom, a common space) according to where they felt most comfortable. Interviews were completed by the first author and a research assistant, trained in cognitive interviewing and administration of the SMMSE with older adults. A family member or friend could be present for the interview duration if the participant preferred.

Once formal, written consent had been received, residents were asked to provide some basic socio-demographic information. The interviewer then completed the SMMSE with the participant. Participants were handed a “practice survey” with three questions with Likert-type scale response options and asked to think-aloud while responding. Following this, participants were asked to complete the paper-based version of the EQ-5D-5L and the EQ-VAS and were encouraged to think-aloud while doing so (concurrent think aloud). If participants were silent for a period of over three seconds, they were prompted to keep thinking aloud (either with scripted or unscripted prompts). If a participant completed a question on the EQ-5D-5L without thinking aloud, they were asked to stop and go back over their responses while thinking aloud (retrospective think aloud). Scripted verbal probes such as “can you tell me what you were thinking about when you were answering that question” and “why did you choose that particular response” were utilised to engage the participant in retrospective think-aloud. The think-aloud portion of the interviews were audio recorded and transcribed verbatim.

### Analysis

Using NVivo qualitative analysis software (release 1.3) sections of text from transcripts were coded to each of the EQ-5D-5L dimensions. The text segments were then exported into an excel spreadsheet and coded to a framework developed from the Tourangeau four-stage response model. The Tourangeau response model is based on a cognitive theory of survey response and identifies four stages at which response issues can occur, comprehension, recall, judgement, and response mapping [18] (Table 1).

**Table 1:** Response model framework

Comprehension	Recall	Judgment	Response mapping
Reflects the encoding process: participants had problems understanding, or a misunderstanding of, a word, phrase or response option OR participants understood the question in a different way than the measure intends the question to be understood (often closely linked to judgement)	Refers to the retrieval process—including using the wrong time frame for retrieval and problems remembering relevant information	Problems with judging the information retrieved, including retrieving irrelevant information or assessing the information inadequately—leading to under or over-reporting of health state (often closely linked to comprehension)	Problems with the response categories including not liking the options or inappropriately applying them e.g., choosing two or not wanting to choose any OR stated answer (verbal protocol data) misaligned with chosen answer (survey response data)

Text segments were independently coded by three authors (KL, MC and LE), with each author keeping notes on their interpretation of the response issue. Coders were blinded to participants’ cognition subgroup and all other demographic details. Inter-coder reliability was estimated using Krippendorff’s alpha (α) [19] and disagreement resolved through a group discussion comprising all three coders. Following coding for response issues, a chi square test was performed to test for association between cognition and response stage issue and frequency, both overall and by dimension. Summary statistics were calculated for socio-demographic data and Pearson’s chi-squared test with Yates’ continuity correction and Kruskal–Wallis rank sum test used to test for association between cognitive impairment subgroup and socio-demographic factors. Pearson’s chi-squared test and Mann–Whitney U were used to test for differences in frequency of response issues by socio-demographic factors (gender, age and level of education). EQ-5D-5L scores were converted to utilities using the Australian pilot value set [20].

## Results

### Participants

Fifty-three older aged care residents expressed interest and gave initial verbal consent. Of these, 50 provided written consent and 46 completed the think-aloud interview; 25 participants with no cognitive impairment and 21 with mild or moderate cognitive impairment (Table 2), slightly exceeding the initial target of 20 per subgroup. Participants were mostly female (65%) with a mean age of 86.4 years. Participants in the cognitive impairment (CI) subgroup were more likely to be older and female than those in the no cognitive impairment (NCI) subgroup (Table 2). There was a statistically significant association between education level and cognitive impairment, χ^2m^ = 8.68, *p* = 0.04, with higher education level being associated with no cognitive impairment. No statistically significant differences were detected for age or other sociodemographic factors by cognition sub-group.

**Table 2:** Participant Characteristics

Cognitive group (SMMSE)	No cognitive impairment	Mild/moderate cognitive impairment	Total
Total	25	21	46
*Age*			
Mean (SD)	84.8 (9.12)	88 (7.54)	86.4 (8.46)
Median (25th & 75th percentiles)	85 (79, 92)	90 (83, 92)	86.5 (80, 92.2)
*Gender: n (%)*			
Female	13 (52.00)	17 (80.95)	30 (65.22)
Male	11 (44.00)	4 (19.05)	15 (32.61)
Education: n (%)*			
Primary school	3 (12.00)	8 (38.10)	11 (23.91)
Some secondary school	12 (48.00)	12 (57.14)	24 (52.17)
Completed secondary school	3 (12.00)	0 (0.00)	3 (6.52)
Tertiary (vocational or university)	6 (24.00)	1 (4.76)	7 (15.22)
*Living resi. care: n (%)*			
< 12 m	5 (20.00)	5 (23.81)	10 (21.74)
1–3 y	9 (36.00)	7 (33.33)	16 (34.78)
≥ 3 y	9 (36.00)	7 (33.33)	16 (34.78)
*Birth country: n (%)*			
Australia	21 (84.00)	18 (85.71)	39 (84.78)
England	1 (4.00)	2 (9.52)	3 (6.52)
Other	2 (8.00)	1 (4.76)	3 (6.52)
*Location: n (%)*			
Metropolitan	3 (12.00)	2 (9.52)	5 (10.87)
Regional	22 (88.00)	19 (90.48)	41 (89.13)
*EQ-5D-5L Utility score:*			
Mean (SD)	0.440 (0.372)	0.468 (0.422)	0.453 (0.391)
Median (25th & 75th percentiles)	0.498 (0.224, 0.704)	0.597 (0.255, 0.723)	0.5 (0.232, 0.718)
*EQ-5D-5L VAS score:*			
Mean (SD)	64.2 (26.4)	72.4 (20.7)	67.9 (24)
Median (25th & 75th percentiles)	70 (50, 81.2)	77.5 (50.8, 90)	75 (50, 86.2)

*P > 0.05

### EQ-5D-5L responses

Slightly higher utility and VAS scores were observed for the CI subgroup than for the NCI subgroup. Whilst there was a difference between cognition sub-groups in mean and median scores for both the VAS and the five dimensions, none were statistically significant.

### Quantitative analysis of response issue data

#### Overall issues

Prior to the group discussion, inter-coder reliability from independent coding was estimated at 70% (α = 0.4945). Following discussion, consensus was reached on the categorisation of all response issue data. Response issues were identified across all stages of the response model, all questions and both cognition subgroups. However, it was apparent that some EQ-5D-5L dimensions led to more significant response issues than others. The usual activities dimension was associated with the highest quantity of response issues (*n* = 40).

#### Response issues by Cognition sub-group and socio-demographic factors

Chi square tests showed that there was no association between cognition and total issue frequency across most dimensions and all response stages. The exception was for the mobility dimension where a significantly greater proportion of total issues was recorded for the NCI subgroup χ^2^(2, *N* = 46) = 9.059, *p* = 0.011. Furthermore, within the mobility dimension, this association was only significant for the comprehension response stage χ^2^(1, *N* = 46) = 5.022 (*p* = 0.25). Pearson’s Chi -square and Mann–Whitney-U tests showed no significant differences in response error frequency by age, education or gender.

#### Response issues by dimension

Table 3 details the response issues by dimension and cognition subgroup. There was a high number of response issues on the usual activities dimension for the comprehension and judgement response stages (38 and 30% of within dimension response issues respectively). There was also a high co-occurrence of judgement and comprehension issues within this dimension (73% of participants who had a comprehension issue also had a judgement issue). Response stage issues varied by dimension in terms of the frequency of issues in each stage by cognition group. For the pain/discomfort dimension for example, recall issues were more prevalent in the CI subgroup and response mapping issues more common in the NCI subgroup whilst for the personal care dimension the reverse was found.

**Table 3:** Quantity response Issues by dimension and cognitive group

	Mobility		Personal care		Usual activities		Pain and discomfort		Anxiety and depression		EQ VAS	
	NCI	CI	NCI	CI	NCI	CI	NCI	CI	NCI	CI	NCI	CI
Comprehension	10	3	3	4	11	4	1	1	1	1	1	3
Judgement	0	1	3	4	8	4	1	0	2	1	2	3
Recall	2	0	3	2	1	4	3	5	4	4	1	0
Response Mapping	5	1	1	5	3	5	5	2	4	1	2	1
Total no. of issues	17	5	10	15	23	17	10	8	11	7	6	7
% of participants who had at least 1 issue	15/25	4/21	6/25	10/21	12/25	11/21	6/25	7/21	6/25	6/21	5/19	4/15
	60%	19%	24%	48%	48%	52%	24%	33%	24%	29%	26%	27%

*NCI* no cognitive impairment, *CI* mild/moderate cognitive impairment—VAS think-aloud data was recorded for *n* = 34

### Qualitative analysis—response issues

#### Mobility

Comprehension response issues within the mobility dimension were principally about whether to factor mobility aids into responses. Some participants assessed their mobility by considering how easily they could walk around without walking aids. However, other participants assumed the question was designed to elicit information about their ability to get around *with* their mobility aids as demonstrated in the example quote included in Table 4.

**Table 4:** Example quotes—issues with dimensions

Dimension	Example quote
Mobility	*I do use a walking frame if I’m going any distance, but otherwise just walking around, no, no problems. Probably to include distance as well as a short thing, you’d have to use the slight problems, simply because if I go any distance, yes, I use a frame. So, how do you qualify it?* (Participant 01*,* NCI Group—participant selected slight problems)
Personal Care	*The second one. Slight problems washing or dressing myself. Well, the girls help me have a shower. But of course, they help me get dressed too, but I can do that generally. I can put myself to bed without their help.* (Participant 36—NCI Group—participant selected slight problems)
Usual Activities	*I have no problems doing my usual activities. Well, my usual activities is mostly in this chair. Oh god, I don’t know how I answer that one. E.g., work, study, housework, family or leisure activities. Well, I don’t do any of those, being in here..* (Participant 29- NCI Group—participant selected unable)
Pain/Discomfort	*I have this pain, discomfort. That comes and goes. I can say I have slight pain or discomfort all the time but then I can round and the next five minutes and go into severe pain because I have various problems. So, I’m not quite sure what to put there. I’m on high pain [medication], sort of thing, anyway. I’ll put moderate.* (Participant 12-NCI Group—participant selected moderate problems)
Anxiety/Depression	*I’m not anxious or depressed. I am slightly anxious or depressed. I am moderately anxious or depressed. I am severely depressed. I wouldn’t consider myself depressed. I do get anxious on the odd occasion, so what will I do there? Cross out depressed, and put slightly anxious*. (Participant 30—NCI Group—participant selected slight problems)
EQ-VAS	*So the worst—it’s like, my health is bad. I know that. But that doesn’t—it does depress me, but I don’t sit and think—I know I’ve got to put up with it, and there’s nothing that can be done about it…Well, I suppose I can’t say I have the worst health. Because people have worse health than I have and are in more pain. And I would say, just to be fair, I’d say 50%. I realise I’m—I know people who are in far worse health than I am.* (Participant 02—CI Group)

#### Personal care

As with the mobility dimension and the use of mobility aids, some participants were confused about how to factor the assistance they receive when washing and dressing themselves into their response. Similarly, some participants responded according to what they believed they could do by themselves instead of what they *actually did*. This type of response, illustrated with the example quote in Table 4 was coded as a judgement issue.

#### Usual activities

Comprehension issues for usual activities were most often related to the examples provided in the question (work, study, housework, family or leisure activities). Participants showed a tendency to use these examples to judge what information they should factor into their response. The examples listed, however, were not activities related to the everyday lives of most respondents which confounded some respondents by drawing them to information related to their lives before moving into residential care and prompting them to formulate responses not based on the recall period of ‘today’. This type of response accounted for the high co-occurrence of comprehension and judgment issues on this dimension.

#### Pain/discomfort

Issues coded to the recall and response mapping stages for this dimension, were commonly coded as such due to consideration of fluctuations in pain levels and the use of pain relief medication or other pain treatments. Response issues were coded to the recall stage when it was explicitly clear that participants were considering a time frame *other than today,* often evidenced by the participant considering information in a general sense or referring to fluctuations in their health state. Whilst this implies that participants were attempting to formulate a meaningful response that allowed for fluctuations, as the EQ-5D-5L asks exclusively about the respondents’ health *today*, these responses were nevertheless considered a recall issue. As in the tabled example, there were a few occasions overall (but particularly with this dimension) where participants chose ‘the middle option’ to avoid a possible misrepresentation involved with answering the question in the “today” timeframe. Where comprehension or judgement issues were found, they related to the inclusion of pain relief regiment into the formulation of the answer.

#### Anxiety/depression

The primary issue for this dimension arose from the participants having to consider the combination of two constructs (anxiety AND depression) within the dimension. Some participants expressed a preference for two different response options to reflect the two health states as in the tabled example quote.

#### EQ VAS

In contrast to the five EQ-5D-5L dimensions, participants appeared to be more aware of the word *today* when reading this question, with only three participants clearly assessing information from a divergent recall period. The participant quoted in Table 4 demonstrated the most common response issue, which was the comparison of their current health state with others of the same age or with what one might expect to experience, rather than their health in comparison to *the best health they could imagine*, as the question asks. This was coded as reflective of both judgement and comprehension issues.

## Discussion

The findings from this study indicate that contextual factors, specific to older adults, may affect the reliable and consistent comprehension of the EQ-5D-5L. A high frequency of response issues were evident across all dimensions and across both cognition groups. Previous think-aloud research applying the Tourangeau model to assess responses to the EQ-5D-3L in the adult general population found a far smaller proportion of response issues, with just 15 response issues across 34 participants [21], indicating that this general population sample largely correctly interpreted the EQ-5D-5L descriptive system as expected by the instrument developers. However, a study that used the Tourangeau model to analyse think-aloud responses to the EQ-5D-3L in a population of community dwelling older adults, found a response issue to participant ratio comparable to the current study, 33 issues across 10 participants [22]. This suggests that interpretation of the EQ-5D for both the three and five levels versions may be problematic for older adults.

It was evident that aged care residents may interpret and respond to the EQ-5D-5L in a way that general population samples may not. The ubiquitous use of treatments or aids in this population and confusion about how to incorporate these factors when formulating a response was responsible for several response issues across three dimensions. In the current study, the examples provided in the ‘usual activities‘ dimension were largely unrelated to life in a residential aged care facility, leading to confusion about what ‘usual activities‘ to consider. The same response issue was also reported in van Leeuwen’s [22] sample of community dwelling older adults.

Participants reported having no problems or slight problems with some dimensions, particularly personal care, despite receiving daily assistance with personal care tasks or, in the case of the mobility dimension, almost exclusively using mobility aids. It is common for mobility-impaired individuals to reframe mobility items to better reflect their situation, including the use of aids [23, 24]. This finding also aligns with early research on using cognitive interviewing to develop surveys for older adults [25]. The authors found that older adults reported an ability to complete tasks despite not having attempted them for weeks or months, and despite reportedly struggling with the tasks previously.

The reluctance of participants to incorporate two distinct concepts into responses to the anxiety/ depression dimension supports the findings of previous research in a similar population [26]. Whilst this question structure has been found to be particularly problematic for people with cognitive impairment, in our study, issues with the combining of two concepts was also observed in the NCI sub-group. Difficulties or discomfort with these questions may also be exacerbated in an aged care population where there may be generational stigma regarding acknowledging mental health conditions, particularly depression [25–28].

Participants attempted to formulate meaningful responses where possible as demonstrated by the adoption of strategies to overcome the problem of fluctuations and combined concepts. These strategies included assessing their health over a longer time-period (‘averaging’), choosing the ‘middle option’, or crossing out part of the question e.g., indicating that their response only related to discomfort or anxiety, not both. The problem of fluctuating health states, particularly on the pain/discomfort dimension has been observed in a diverse range of populations, including patients with cancer [29], patients with advanced HIV [30], and patients nearing death [31]. The same pattern was also identified in a study examining the feasibility of using the EQ-5D-3L with nursing home residents [32]. Whilst some commentators have suggested that recall periods of a week or more may mitigate this [33, 34], it is acknowledged that longer recall periods can be problematic, especially for people with cognitive impairment [35]. Furthermore, respondents with widely fluctuating health and/or QOL may equally struggle with longer time frames [36].

Older adults, particularly those with functional disability or long-term health conditions are likely to show what is termed ‘Beta’ response shift or ‘scale recalibration’, where a person adapts to declining health over time by shifting their reference point to reflect their lowered expectations of health and/or QOL [21, 37, 38]. Questions that require interpretations of especially subjective concepts such as “health” and “best” are particularly at risk of obfuscation from Beta response shift effects [39]. This was particularly evident in our study by participants’ responses to the VAS, which revealed that some participants compared themselves to others of the same age or to what they might expect their health to be given their age. Unsurprisingly, individuals with lowered expectations under-report health-related problems [40], as demonstrated by the tendency of participants in this study to adjust their responses to reflect what they deemed to be a realistic expectation of health for their age or circumstance.

Although the EQ-5D has been psychometrically tested in older people, including dementia populations, and found to be feasible [41, 42] and reliable [43, 44], our findings raise questions as to the content validity of the EQ-5D-5L with an older aged care resident population. To ensure the EQ-5D-5L is meaningful for this cohort, some alterations to the phrasing may be necessary. Modifications, including providing more relevant examples of ‘usual activities‘, explicit clarity around what constitutes ‘best health’, wording that allows for, and explains further how residents should factor fluctuating health and specific instructions on how to factor mobility aids and other treatments, may increase the utility of the EQ-5D-5L with an aged care and older person specific population. Consideration should also be given to the appropriateness of the ‘today’ timeframe for this cohort or to rephrasing questions to place more emphasis on instructions for the recall period.

Regardless of the measure adopted, assessing QOL in older people in residential care settings is challenging due to the relatively high prevalence of cognitive impairment and dementia in this population. To drive inclusivity in self-reporting of QOL wherever possible, some instrument developers have developed easy-read and pictorial versions that have been tested with older adult service care recipients and found to have increased comprehensibility [44, 45]. It has also been suggested that support from an interviewer may be necessary to ensure meaningful responses to QOL measures in older people with cognitive impairment [46]. Towers and colleagues [47] have developed a mixed methods version of the Adult Social Care Outcomes Toolkit (ASCOT) and tested its acceptability and feasibility to inform practice in UK care homes. It was found that the mixed methods approach directly facilitated inclusion of many cognitively impaired residents who otherwise would have been excluded from the research or had their own views on their social-care related QOL represented only by a proxy.

This study has several limitations. Whilst the aim was to recruit even numbers in each sub-group, COVID19 pandemic related recruitment barriers at the time of data collection and aged care facilities’ tendency to recommend participants with no cognitive impairment contributed to uneven sub-group numbers. Interviewers were vigilant to reduce or change prompts when participants became fatigued or frustrated, nevertheless some participants clearly struggled with the think-aloud process and this may have caused them to lose focus and attend less carefully to their responses. The administration of the SMMSE immediately prior to the think-aloud interview may have further exacerbated this issue by adding to the cognitive load and thereby fatigue. There is also some evidence that the added cognitive burden of the think-aloud protocol can contribute to this effect [34, 48]. Data was not collected on the use of mobility aids, personal care assistance and treatments or medications for this sample. Controlling for these factors may have provided more clarity around the patterns of response issue across cognition sub-groups. Finally, whilst the completion rate for this study was high, this cannot be relied upon to reflect the self-report feasibility of this measure within this population due to the involvement of interviewers in the completion process. Despite these limitations this study provides valuable insights and to our knowledge represents the first in-depth qualitative study in Australia and internationally to apply a think-aloud protocol to improve our understanding of how older residents with different levels of cognitive impairment interpret and respond to the EQ-5D-5L descriptive system. The findings have potential wider relevance and application beyond the EQ-5D-5L for the development and application of other QOL tools administered with populations of older people.

## Conclusions

This study to examine aged care residents’ responses and interpretations of the EQ-5D-5L has highlighted several response issues of relevance to this population including, how to factor in the use of mobility aids, pharmaceuticals and care services into responses as well as potentially frequent fluctuations in health and/or QOL over time. The inclusion of relevant contextual examples and explicit instructions (e.g., relating to factoring in mobility aids, health treatments and fluctuating health states) may promote instrument completion more closely aligned with the underlying EQ-5D-5L concept model for this cohort.

### Supplementary Information

Below is the link to the electronic supplementary material.

Supplementary file1 (PDF 143 KB)

Supplementary file2 (PDF 255 KB)

## Data Availability

Data are not publicly available due to Flinders University ethics requirements but are available from the corresponding author on reasonable request.
