# Supplementary material for: How do older adults receiving aged care services understand and respond to the EQ-5D-5L? A think-aloud study in residential care
Source: Qual Life Res. 2023 Jun 29;32(11):3161–70. doi: 10.1007/s11136-023-03466-2 (PMC10522713; doi:10.1007/s11136-023-03466-2)
Supplement: Supplementary file 1 — Supplementary file1 (PDF 143 KB) [file 11136_2023_3466_MOESM1_ESM.pdf]

How do older adults receiving aged care services understand and respond to the EQ-5D-5L?

A think-aloud study in residential care

Quality of Life Research

Kiri Lay[1], Matthew Crocker[1], Lidia Engel[2], Julie Ratcliffe[1,3], Simon Charlton[4], Claire Hutchinson[1,2]

[1]Health and Social Care Economics Group, Caring Futures Institute, Flinders University, Australia

[2]Health Economics Division, School of Public Health and Preventive Medicine, Monash University, Melbourne, Victoria, Australia

[3]Caring Futures Institute, College of Nursing and Health Sciences, Flinders University, Australia

[4]South Australian Innovation Hub [address details]

Corresponding Author: Kiri Lay, Health and Social Care Economics Group, Caring Futures Institute, Flinders University, Adelaide, GPO Box 2100, Adelaide, SA 5001, Australia. E: kiri.lay@flinders.edu.au

Online Resource 1. Think-aloud approach

Think-aloud is a qualitative approach rooted in cognitive psychology, designed to elicit the thought processes of the participant [1]. The approach can be usefully applied to gain insights into participants' survey response processes e.g., in responding to the EQ-5D-5L, to provide evidence of the level of comprehension and understanding. Think-aloud has been used previously with older people with dementia to examine the decision-making heuristics involved in responding to Discrete Choice Experiments (DCEs) [2] and in the development of the DEMQOL, a dementia specific measure of QOL [3, 4].

Contemporary think-aloud protocols for survey testing can include one or (more commonly) a combination of the following methods: the participant is asked to "think-aloud" while completing the survey task (concurrent think-aloud), the participant is asked to review their responses and explain their thought processes after each question or at the conclusion of the survey (retrospective think-aloud), and/or the participant is prompted to share their thoughts on a question, portion of a question or the survey as a whole (verbal probing) with the aim to elicit specific data on the subject of interest. The use of think-aloud protocols is now recognised as a key tool in survey validation and is recommended in the development of measures [5-7]. In the context of established QOL instruments such as the EQ-5D-5L, think-aloud can help determine if items and response options are being interpreted as intended by the instrument developers [8, 9].

References

1. Ericsson, K.A., *Protocol analysis : verbal reports as data*. Rev. ed, ed. H.A. Simon. 1992, Cambridge, Mass.: Cambridge, Mass. : MIT Press.
2. Hutchinson, C., et al., *Using discrete choice experiments to elicit the service preferences of people with mild intellectual disability: An exploratory study.* Health Soc Care Community, 2022. **30**(4): p. e1396-e1405.
3. Phillipson, L., et al., *A Cohesive Research Approach to Assess Care-Related Quality of Life: Lessons Learned From Adapting an Easy Read Survey With Older Service Users With Cognitive Impairment.* International Journal of Qualitative Methods, 2019. **18**.
4. Smith, S.C., et al., *Measurement of health-related quality of life for people with dementia: development of a new instrument (DEMQOL) and an evaluation of current methodology.* Health Technol Assess, 2005. **9**(10): p. 1-93, iii-iv.
5. Boness, C.L. and K.J. Sher, *The case for cognitive interviewing in survey item validation: A useful approach for improving the measurement and assessment of substance use disorders.* Journal of studies on alcohol and drugs, 2020. **81**(4): p. 401-404.
6. Scott, K., O. Ummer, and A.E. LeFevre, *The devil is in the detail: reflections on the value and application of cognitive interviewing to strengthen quantitative surveys in global health.* Health Policy Plan, 2021. **36**(6): p. 982-995.
7. Collins, D., *Pretesting Survey Instruments: An Overview of Cognitive Methods.* Qual Life Res, 2003. **12**(3): p. 229-238.
8. Phillips, A.C.E., *The Usefulness of 'Think-Aloud' for Evaluating Questionnaires in Use in the Health Domain*. 2014, ProQuest Dissertations Publishing.
9. Engel, L., et al., *A qualitative exploration of the content and face validity of preference-based measures within the context of dementia.* Health Qual Life Outcomes, 2020. **18**(1): p. 178.
