# Supplementary material for: How do older adults receiving aged care services understand and respond to the EQ-5D-5L? A think-aloud study in residential care
Source: Qual Life Res. 2023 Jun 29;32(11):3161–70. doi: 10.1007/s11136-023-03466-2 (PMC10522713; doi:10.1007/s11136-023-03466-2)
Supplement: Supplementary file 2 — Supplementary file2 (PDF 255 KB) [file 11136_2023_3466_MOESM2_ESM.pdf]

How do older adults receiving aged care services understand and respond to the EQ-5D-5L?

A think-aloud study in residential care

Quality of Life Research

Kiri Lay[1], Matthew Crocker[1], Lidia Engel[2], Julie Ratcliffe[1,3], Simon Charlton[4], Claire Hutchinson[1,2]

[1]Health and Social Care Economics Group, Caring Futures Institute, Flinders University, Australia

[2]Health Economics Division, School of Public Health and Preventive Medicine, Monash University, Melbourne, Victoria, Australia

[3]Caring Futures Institute, College of Nursing and Health Sciences, Flinders University, Australia

[4]South Australian Innovation Hub [address details]

Corresponding Author: Kiri Lay, Health and Social Care Economics Group, Caring Futures Institute, Flinders University, Adelaide, GPO Box 2100, Adelaide, SA 5001, Australia. E: kiri.lay@flinders.edu.au

Online resource 2: Scripted Interview Guide

INTERVIEW GUIDE
(For residents)

Title: Unravelling the EQ-5D-5L self-report proxy conundrum for older people

Stage 1: Demographic questions

Good morning/afternoon (name of resident), thank you for agreeing to be a part of our study. We hope that through your participation, we will be able to better understand how we can more accurately measure your quality of life.

For the first part of the interview, I will ask you some simple questions about yourself. There are 7 questions in total. I will sit next to you and we can answer them together.

(If the resident is able, allow them to answer each question independently. If they appear to need help, offer to read the questions and record their answers for them)

Unravelling the EQ-5D-5L (Residents)

1. Participant ID

Enter your answer

2. What is your date of birth?

Please input date in format of M/d/yyyy

3. Gender

- ◯ Woman
- ◯ Man
- ◯ Non-binary
- ◯ Prefer not to say

4. What was your country of birth?

Enter your answer

5. Is English your first language?

- Yes
- No

6. What was the highest level of education you obtained?

- Primary school
- Some secondary school
- Completed secondary school
- Vocational training
- Undergraduate university
- Postgraduate university

7. How long have you lived in residential care?

- <6 months
- 6 - 12 months
- 1 - 2 years
- 3 - 5 years
- 6 - 10 years
- > 10 years

Screening stage:

Before you take part in the interview stage of the research, I would like to ask you some simple questions. The questions I will ask are designed to measure your level of cognition. You may find some of the questions hard, which is fine. All I ask is that you do your best.

Your answers will not be shared with anyone else and your level of care will be unaffected by how you answer. The purpose of the questions are to place you in groups of other aged care residents who have a similar level of cognition.

Do you have any questions? Are you happy to commence?

(Follow the SMMSE protocol)

OFFER Break to Participant

Stage 2: EQ-5D-5L

Thank you very much. Next I will give you a survey to answer. There are 6 questions which are designed to assess your quality of life. We would like you to take your time to answer each question.
Whilst you complete the survey, I would like to record your thoughts. To do this, I will ask you to think aloud your thoughts as you read and answer each question. As you read each question and each possible option, speak aloud your thoughts. I am interested in what you are thinking when you answer this survey. I want you to tell me everything you are thinking as you read each question and decide how to answer it. I would like you to talk constantly. I do not want you to plan out what you say or explain to me why you are saying it. If you are silent for any period of time I will prompt you with the question 'what are you thinking at the moment'. Again, there is no right or wrong thing to say. Do you have any questions? Are you happy for me to record what you say? (if yes, set up Dictaphone and back-up recording device)

So that you can practice thinking aloud, we'll first ask you to complete a practice survey. Remember to speak aloud your thoughts as you consider and respond to each question.

Hand over practice survey and pen.

We will start the survey now. Remember to think aloud your thoughts as you read and answer each question.

If the resident is not verbalising their thoughts after the first question, prompt the resident to verbalise their thoughts. Suggested prompts (avoid 'why' if possible)
What are you thinking at the moment?

If participant is still not verbalising prompt after each question. E.g.

Can you just pause there and tell me why you responded that way? What were you thinking about when you responded that way?

Remember to think aloud as you move onto the next question.
What were your thoughts as you chose that option?
What were your thoughts as you rejected the other options?

Could you please think about your last choice(s) and describe for me what you were considering when you made those choices?

After participant has finished survey

Thank you (name) for volunteering to be a part of our study.

We will collect all the responses from this study and anonymise them so that your responses cannot be identified. We do this to protect your privacy as well as the privacy of the other participants. We will then pool the responses so that the results reflect the average response of all who participated in the trial. We hope to have the results of this study available by the end of next year, but because we will anonymise the responses, we will not be able to provide you individual feedback. Would you like to be informed of the results when they become available?

If yes

We will send a copy of the results to your facility manager when they have been reported and we will ask your facility manager to pass on the results to you.
